# Combination of Biogas Residues and *Bacillus* Interactions Stimulates Crop Production and Salinity Tolerance in *Sorghum bicolor*

**DOI:** 10.1155/sci5/2123395

**Published:** 2024-12-11

**Authors:** Muhammad Waseem Abbasi, Naveed Hussain, Marium Tariq, Muhammad Qasim, Qu Wei, Jianbin Guo, Shoujun Yang, Renjie Dong, Zainul Abideen, Mohamed A. El-Sheikh

**Affiliations:** ^1^Department of Botany, University of Karachi, Karachi 75270, Pakistan; ^2^Yantai Institute, China Agricultural University, Yantai 264670, Shandong, China; ^3^M.A.H. Qadri Biological Research Centre, University of Karachi, Karachi 75270, Pakistan; ^4^Department of Agricultural Engineering, China Agricultural University, Qinghua Donglu 17, Haidian District, Beijing 100083, China; ^5^Dr. Muhammad Ajmal Khan Institute of Sustainable Halophyte Utilization, University of Karachi, Karachi 75270, Pakistan; ^6^College of Agriculture, University of Al Dhaid, P. O. Box 27272, Sharjah, UAE; ^7^Botany & Microbiology Department, College of Science, King Saud University, P.O. Box 2455, Riyadh 11451, Saudi Arabia

## Abstract

Stress tolerance in cereal crops like Sorghum is important to address food security and land development for saline agriculture. Salinity is considered one of the most devastating abiotic stresses affecting plant growth and yield, specifically in water-scared areas of the world. Biogas residue is a good source of plant nutrients with enriched fertilizer for crop yield and productivity. In this study, seeds were sown in the soil supplied with biogas residues (0% and 5% w/w). After seedling establishment, three *Bacillus* strains (B26, BS, and BSER) were introduced around the roots of Sorghum. Saline water irrigation started after a week of bacterial inoculation. Sorghum plants were uprooted after 30 days of saline water irrigation. Results indicated that the *Bacillus* strain and biogas residues showed the highest plant growth in both (0 and 75 mM) salinity levels. Further, this *Bacillus* strain modulated Sorghum's secondary metabolites (phenols and flavonoids) and osmoprotectants (proline and soluble sugars) under salinity stress. Reduction in salinity stress demonstrated lower activities of antioxidant enzymes including catalase, ascorbate peroxidase, and superoxide dismutase; however, guaiacol peroxidase activities were enhanced in *Bacillus* (BS strain) treated plants with biogas residues application. Among the three strains, BS strain demonstrated better results with biogas residues under salinity stress in *Sorghum bicolor*.

## 1. Introduction

Sorghum (*Sorghum bicolor* L. Moench) is a Poaceae family cereal grain plant. The plant likely originated in Africa, where it is a major food, feed, forage crop, and a staple food for millions of poor people [1]. It is vital to make starch, fiber, dextrose syrup, alcohol, and biofuels. In most countries, this crop is grown for fodder and grains and is considered an essential feed for livestock to maintain excellent cattle health. It is grown in Pakistan on an area of 172 per thousand hectares with an average yield of 0.7 tons per hectare [2]. Sorghum is a strong grass and usually grows to a height of 0.6–2.4 m (2–8 feet), sometimes reaching as high as 4.6 m (15 feet). This crop is vulnerable to various diseases like turcicum leaf blight, downy mildew, anthracnose, loose smut, long smuts, charcoal rot, and leaf spot, resulting in production losses [3–6]. Further, various abiotic stressors, including drought and salinity, limit the growth and yield of Sorghum in different areas of the world.

Salinity is considered one of the most devastating environmental stresses that drastically curtails the productivity and quality of crops worldwide. More than 20% of the world's cultivable lands are dealing with the adversity of salt stress, and these salt-prone areas are continuously increasing due to both natural and anthropogenic activities. However, this adversity has become much more severe in arid and semiarid regions over the last 20 years due to increasing demand for irrigation water requirements [7]. Pakistan, being an agricultural country, has a well-developed irrigation system. In contrast, poor irrigation water quality converted agricultural lands into saline areas, estimated to be up to 50% globally, and 14% of irrigated land in Pakistan has declined due to salinity [8]. Increased concentration of salts in soil results in soil degradation with poor plant growth, low osmotic potential of soil, hormonal imbalance, ion toxicity, susceptibility to diseases, and physiological drought [9, 10].

Several strategies are available for the remediation of salinity-affected soil, like chemical remediation and bioremediation, and due to these approaches, plants can survive under salinity stress with optimum production [11]. Some chemicals have been found to stimulate the secondary metabolism of plants to provide some strength against salinity stress [9, 12]. In areas where the climate is semi-arid to arid, and the soil has a high pH, remediation can be done by the combination of salt-tolerant microbes along with organic amendments to improve soil fertility and health [10]. Studies regarding Plant growth promoting rhizobacteria (PGPR) assistance are increasing rapidly to acquire commercially low-cost and environmentally safe crop management [13]. Various mechanisms are reported for the growth promotion of different types of crops like phytohormone production, nitrogen fixation, phosphorus solubilization, biocontrol agents, and induction of systemic resistance for managing plant pathogens attack [14–18]. Many species from the genus *Bacillus* are very well-known PGPR because of their ability to colonize plant tissues and produce bacteriocins, antimicrobial peptides, lipopeptides, siderophores for iron acquisition, and polyketides [19–22]. They also exhibit biocontrol abilities by inducing systemic resistance using pathogen-associated molecular patterns and basal defense systems with genes of proteins in the host plant [23, 24]. This helps promote the growth of plants by producing and modulating hormones like gibberellins, auxins, ethylene, and jasmonic acid [25, 26].

Biogas residues (BR) devised from anaerobically digested animal waste are considered a good source of plant nutrients with enriched fertilizer for the establishment and productivity of crops [27, 28]. BR maintains the availability of nutrients in the soil, mineralizes carbon, and nitrogen and improves crop productivity and ecological functions [29, 30]. The application of BR improves the physical and chemical characteristics of the soil and affects the structure and diversity of soil bacterial and fungal communities [31, 32]. Research revealed that applying a higher concentration of biogas slurry produces maximum alpha diversity of the fungal community, particularly for *Aspergillus*, *Trichoderma*, and *Penicillium* species. However, this phenomenon does not apply to bacterial diversity [33]. The effect of biogas slurry in sandy soil was found to be most significant for bacterial communities, which proved that the structure of these communities with lower biomass is more sensitive to organic responses [28]. Another concern about the application of biogas slurry and residues in agricultural soil is an increase in the electrical conductivity of soil. Therefore, by applying different bacterial species, the current research aims to evaluate the effect of the selected concentration of BR from cow manure on Sorghum growth. This combination is used by Sorghum plants to enrich the beneficial microbial load in BR-treated soil and utilize minerals from the soil to reduce the electrical conductivity before saline water irrigation. In this study, greenhouse experiments were conducted on Sorghum. Salinity stress was also applied to record its effect on the physiology of plants. We hypothesized that combining biogas slurry and bacterial strains would combat salinity stress and reduce the toxic effect on sorghum seeds.

## 2. Materials and Methods

### 2.1. Collection of BR

The biogas slurry was provided by a scale production plant, where cow manure was the main raw material for anaerobic digestion. The digestion process was set at a temperature of 35°C, and the retention time was almost two weeks. The biogas slurry contained dry matter (1.5%–2.4%), pH (7.50–7.65), total phosphorus content of 71 mg/L, total potassium (K) content of 85 mg/L, total nitrogen content of 1023 mg/L, and electrical conductivity of 3.1 mS/cm, respectively. Biogas slurry was dried under sunlight to evaporate water and the BR were collected and applied to the experimental soil.

### 2.2. Bacterial Isolates

Three *Bacillus* strains including B26 (accession number PQ326446), BS (accession number OQ799655), and BSER (accession number OQ799400) were isolated from the rhizosphere of different plant species. These strains were identified based on 16s rRNA gene amplification. These strains were chosen because of their ability to improve biotic stress resistance in different plants [14]. *Bacillus* species previously showed adaptation to salt and drought stresses [34, 35] and the population count on NaCl showed a population in salt stress as 3.6 ∗ 10^9^, 8.1 ∗ 10^9^, and 4.5 ∗ 10^8^ CFU/mL, respectively.

### 2.3. Experimental Design and Treatments

A pot experiment was conducted to observe the effect of the combined use of BR and *Bacillus* strains on the growth and physiology of Sorghum under different salinity levels. Pots were arranged in the greenhouse using a completely randomized design (CRD). The seeds of *Sorghum bicolor* (L.) Moench (var. SS-77) was purchased from the local market. Seeds were surface sterilized with sodium hypochlorite (1.5% for 5 min) and washed thrice with sterilized distilled water. BR were mixed in the experimental sterilized soil (sandy loam soil) at 0% and 5% (w/w), and five seeds were sown in each pot, which carried 500 g of sandy loam soil. After seedling emergence, only two equal-sized seedlings were maintained in each pot. Ten mL bacterial suspension in each pot (containing two plants) was given after a week of seedling emergence. Two levels of salinity (0 and 75 mM) were selected for this experiment. NaCl solution was prepared in distilled water for irrigation. After 7 days of bacterial inoculation, pots were irrigated with NaCl solution at 0 and 75 mM after every second day and continued for up to 30 days. The treatments included (1) control (without bacteria and no BR (C−)), (2) control (without bacteria and with BR (C+)), (3) bacteria 1 (B26), (4) bacteria 2 (BS), (5) bacteria 3 (BSER), (6) B26 +BR (BR at 5%), (7) BS + BR 5%, and (8) BSER + BR 5%. An adequate moisture level was maintained in the pots by applying sterilized distilled water at regular intervals until harvesting. After harvesting, fresh growth parameters, along with antioxidant enzymes, secondary metabolites, and osmoprotectants, were also evaluated.

### 2.4. Secondary Metabolites

Phenolic compounds were analyzed using the Folin–Ciocalteu reagent method [36], where the reaction mixture was prepared by adding Folin Reagent (0.2 N) with sample extracts/standard and incubated for 5 min. Added sodium carbonate (75 g/L) and then incubated for a further 90 min at room temperature, after which absorbance was measured at 765 nm using a UV-visible spectrophotometer, and the phenolic content was calculated using a calibration curve made by gallic acid.

An aluminum chloride method assesses total flavonoid concentration [37]. Quercetin was dissolved in 50 mL of 80% methanol to make a standard solution of (100 g/mL). It was later diluted to create several calibration standards that connect the absorbance of the solution to its flavonoid level. Aluminum chloride was applied to both the sample and the standards, creating a colorful complex. The absorbance was measured using a UV-visible spectrophotometer at 415 nm, and the flavonoid content was calculated using the absorbance readings. The total flavonoid content (TFC) of the samples was calculated by extrapolating the calibration curve made using the standards for quercetin, allowing correlation between absorbance values and flavonoid levels. The results were expressed as milligram Quercetin equivalents per gram.

### 2.5. Osmoprotectants

Soluble sugars were estimated using the anthrone colorimetric method following the method of Zhang et al. [38]. Add 1 mL of extract and 5 mL of 0.2% anthrone, mix by shaking, and now incubate at 80°C for 10 min for color formation. After cooling, the absorbance was recorded at 620 nm. Soluble sugar and starch content were calculated using the following formula:

(1) $$\begin{array}{c} \text{Soluble sugar}\left(\%\right)=\frac{C.n.\left(V/\alpha \right)}{W\times 1000}\times 100\%, \end{array}$$

where *C* is the glucose content (μg) of the tested sample in the cuvette, which was read from a standard curve; *V* represents the total volume of extracts (mL); *α* represents the volume of extracts (mL) used in displaying color; *n* represents the fold of dilution; *W* is the dry weight of the sample (mg). To observe the amount of proline, ninhydrin acid, glacial acetic acid, and a proline solution in the ratio 1:1:1 were incubated at 90°C for an hour and then iced cooled. Adding 2 mL of toluene extracted the chromophore, and the absorbance was recorded at 520 nm [39].

### 2.6. Antioxidant Enzyme Extraction

A fresh leaf sample (1 g) was frozen in liquid nitrogen to prevent proteolytic activity and ground with 10 mL of buffer (0.1 M phosphate buffer with 0.5 mM EDTA for catalase (CAT) and guaiacol peroxidase (GPX) while adding 1 mM ascorbic acid to EDTA for ascorbate peroxidase (APX)). This mixture was filtered using four layers of cheesecloth and cold centrifuged at 15, 000*xg* for 20 min, obtaining supernatant, which was used as an enzyme.

#### 2.6.1. CAT; E.C.1.11.1.6

The amount of CAT enzyme was estimated using the Aebi protocol [40]. The reaction mixture (3.0 mL) has K phosphate buffer (1.5 mL of 100 mM buffer), hydrogen peroxide (12.5 mM), enzyme (50 μL), and water to make up the final volume of 3.0 mL. Hydrogen peroxide was added to initiate the reaction, where a decrease in absorbance (240 nm) was recorded for 1 min. Compared with a standard curve drawn using a known concentration of hydrogen peroxide. CAT activity was recorded using the following formula:

(2) $$\begin{array}{c} \text{Initial reading}-\text{final reading}=\text{quantity of hydrogen peroxide reduced}\left(\min^{-1}\;g^{-1}\text{ fresh weight}\right). \end{array}$$

#### 2.6.2. APX; EC.1.1.11.1

Nakano and Asada [41] used this method. In this method, a reaction mixture (3 mL), K phosphate buffer (50 mM) having pH 7.0, ascorbic acid (0.5 mM), 0.1 mM EDTA, 0.1 mM H_2_O_2_, 0.1 mL enzyme, and adding 0.7 mL water to make final volume upto 3.0 mL. 0.2 mL of hydrogen peroxide was added to initiate the reaction, and absorbance was measured using a UV-visible spectrophotometer at 290 nm. The formula used to calculate APX is as follows:

(3) $$\begin{array}{c} \text{Initial reading}-\text{final reading}=\text{quantity of ascorbic acid oxidized}\left(\min^{-1}\;g^{-1}\text{ fresh weight}\right). \end{array}$$

#### 2.6.3. GPX; EC 1.11.1.7

This activity was measured using the protocol followed by Egley et al. [42]. Add reaction mixture (3 mL), K phosphate buffer (50 mM, pH 7.0), guaiacol (75 mM, H_2_O_2_ 10 mM), and enzyme extract (20 mL). The activity was determined from the increase in absorbance at 470 nm for 2 min.

#### 2.6.4. Superoxide Dismutase (SOD; EC 1.15.1.1)

The activity of SOD was ascertained as described by Beauchamp and Fridovich [43]. The reaction mixture contained phosphate buffer (50 mM), EDTA (0.1 mM), methionine (13 mM), nitroblue tetrazolium (75 μM), enzyme extract (100 μL/3 mL of the reaction mixture), riboflavin (2 μM; added at the end to initiate the reaction in the fluorescent light). After 10 min of exposure, reaction products were measured at 560 nm.

### 2.7. Data Analysis

The experiment was conducted on a greenhouse bench using a completely randomized design. Data were collected and subjected to the Analysis of Variance (ANOVA). The figure shows the mean of five replicates ± standard error of means. Tukey test was performed as a post hoc test to find out if there are significant differences between the mean. Principle component analysis (PCA) was performed on the replicated of all the dependent variables against 16 different treatments (independent variables) of controls, bacteria, and BR with two salinity levels (0 and 75 mM) using the SRplot online tool [44].

## 3. Results

### 3.1. Plant Growth

**Figure 1:**
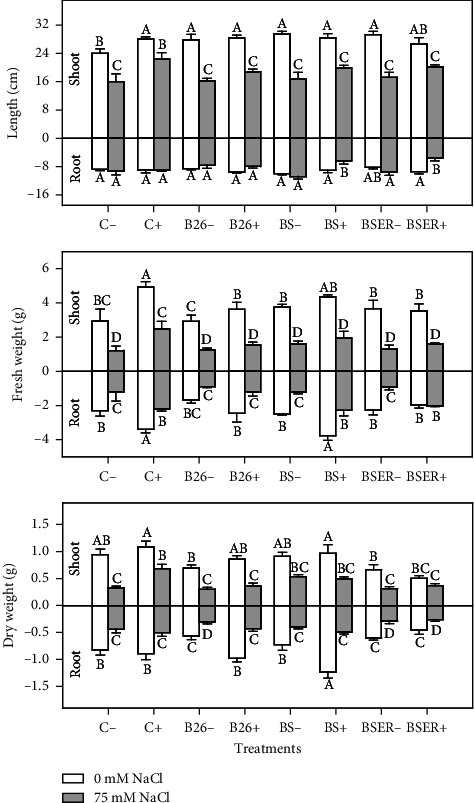
Effect of different Bacillus strains (B26, BS, and BSER) on length, fresh weight, and dry weight of Sorghum shoot and root in the presence (+) and absence (−) of biogas residues, under 0 and 75 mM NaCl. Dissimilar letters on the bars showed significant differences between the means according to Tukey's HSD test.

Based on Figure 1, it was computed that all growth parameters showed a declining response when irrigated with salinity (75 mM). However, plants grown in the soil with BR demonstrated higher shoot and root growth than controls (without BR). The highest shoot lengths were observed in plants treated with strain BS without salinity treatments (BS− with 0 mM) followed by BSER strain (BSER− with 0 mM). Interestingly, Sorghum plants treated with BR and salinity treatments (C+ with 75 mM) demonstrated plant height and fresh weights almost similar to the plants without salinity and BR (C− with 0 mM). Sorghum plants with BR showed higher shoot weights in both salinity treatments (0 and 75 mM). BS strain with BR showed higher shoot weight than other tested strains (B26 and BSER). Root length is considered a vital physical growth parameter, and it was maximum when BS without slurry was inoculated in soil. Similarly, BS with BR (BS+ with 0 mM) demonstrated the highest root weights. All results showed that BS with BR enhanced the growth of the Sorghum plant (Figure 1).

### 3.2. Osmoprotectants

**Figure 2:**
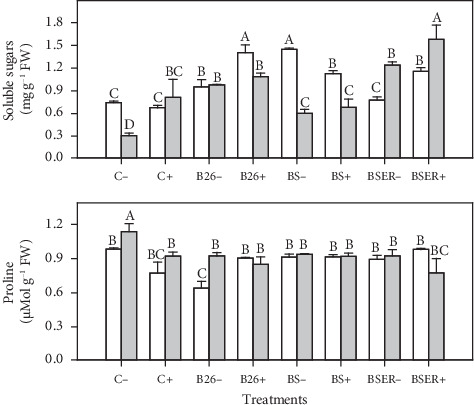
Effect of different *Bacillus* strains (B26, BS, and BSER) on soluble sugars and proline contents of Sorghum in the presence (+) and absence (−) of biogas residues, under 0 and 75 mM NaCl. Dissimilar letters on the bars showed significant differences between the means according to Tukey's HSD test.

High salinity in plants causes osmotic stress which links with the synthesis of organic solutes like sugars and proline in the cytosol and this natural phenomenon is useful to cope with the salinity stress in plants. Bacterial applications on Sorghum in both salinity conditions showed a general increase in the concentrations of soluble sugars. The highest concentrations of soluble sugars were observed in plants treated with BSER strain and BR under saline stress conditions (BSER+ with 75 mM). Control plants without BR (C−) in both salinity treatments (0 and 75 mM) showed higher proline contents in sorghum plants compared to other treatments. In general, proline concentrations in Sorghum increased with the application of BR (Figure 2).

### 3.3. Secondary Metabolites

**Figure 3:**
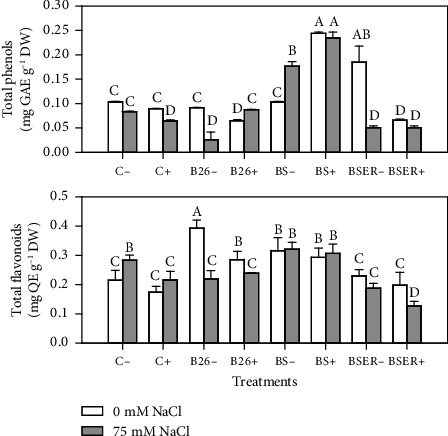
Effect of different *Bacillus* strains (B26, BS, and BSER) on total phenols and total flavonoid contents of Sorghum in the presence (+) and absence (−) of biogas residues, under 0 and 75 mM NaCl. Dissimilar letters on the bars showed significant differences between the means according to Tukey's HSD test.

Total phenolic compounds in Sorghum plants were varied with different treatments of bacteria, BR, and salinity. Sorghum plants treated with BS showed the most significant increase in total phenolic compounds, especially when treated with BR. In controls (C− and C+), the total amount of phenolic contents remained lower with the increase in salinity (75 mM). Flavonoid contents also remained higher in the leaves of Sorghum when treated with BS strain in saline (75 mM) and nonsaline conditions (0 mM). However, BSER showed comparatively lower levels of flavonoid contents. BS strain has an increasing impact on secondary metabolites of Sorghum plants (Figure 3).

### 3.4. Antioxidant Enzymes

Antioxidant enzymes in sorghum plants showed varied responses against the different applied treatments of bacterial strain, BR, and salinity levels. CAT activities in sorghum leaves declined in the plants treated with BR (C+) compared to negative controls (C−). B26 strains in saline and nonsaline conditions exhibited higher CAT activities in the leaves of sorghum compared to other tested *Bacillus* strains and controls. GPX activities were found lower than other antioxidant enzymes (including CAT and APX) in sorghum leaves. GPX activities in Sorghum control plants remained lower in the controls (C− and C+) under different salinity treatments (0 and 75 mM). The highest activity of GPX was observed in the plants that were treated with BS and BR (BS+) under saline conditions (75 mM). APX activities were observed maximum in the control plants under saline conditions (C− with 75 mM). Higher salinity (75 mM) treatment in most of the treatments displayed higher APX activities compared to non-saline plants (0 mM). The lowest activities of APX were recorded in Sorghum plants treated with BS strain under different levels of BR (BS− and BS+) and salinity (0 and 75 mM). SOD activities were found higher in plants treated with 75 mM of salinity compared to control plants that were given 0 mM of salinity. B26 treated plants revealed higher activities of SOD especially in higher salinity stress. Control plant (C+) without salinity stress showed minimum SOD activities in sorghum.

**Figure 4:**
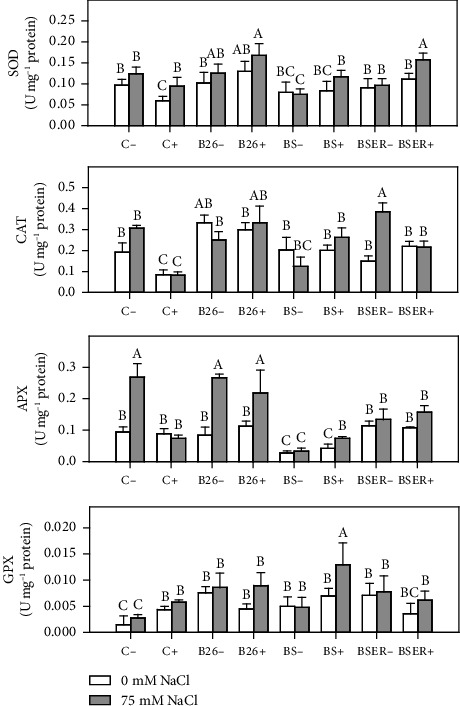
Effect of different Bacillus strains (B26, BS, and BSER) on activities of superoxide dismutase (SOD), catalase (CAT), ascorbate peroxidase (APX), and guaiacol peroxidase (GPX) of Sorghum in the presence (+) and absence (−) of Biogas residues, under 0 and 75 mM NaCl. Dissimilar letters on the bars showed significant differences between the means according to Tukey's HSD test.

**Figure 5:**
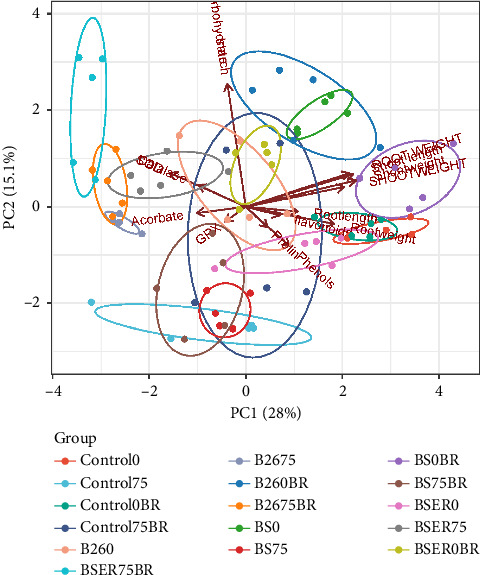
Principal component analysis (PCA) biplot illustrating the variation in Sorghum growth parameters and biochemical traits under different treatments. The plot displays the distribution of Sorghum plants based on different *Bacillus* strains (B26, BS, BSER) and treatment with or without biogas residues (BR) under saline conditions (0 mM and 75 mM NaCl).

Overall, the trend in the experimental data are displayed in Figure 4, indicating that plant growth parameters showed similar results and dispersed together on the PCA plot. Similarly, antioxidant enzymes such as CAT, SOD, and APX showed the same trend as the growth and appeared together in PCA plot6. When treated with BR, Strain BS showed promising results and appeared distinctly on the PCA plot (Figure 5).

## 4. Discussion

Our study revealed the potential role of BR in combination with *Bacillus* strains for induction of salt tolerance in Sorghum. Results showed that BS gained more promising results with BR when applied in soil by improving Sorghum's plant shoot root length and weight. Recently PGPR have been used in combination with biogas slurry to observe the growth of maize in salinity stress [45]. Under high saline conditions, the ethylene level is increased, which becomes condensed due to the treatments of PGPR because some strains consist of ACC deaminase activity for higher plant growth [46]. This ACC deaminase is helpful in water intake from deeper soil in stress conditions. BR were incorporated into the soil before inoculation of bacteria. BR is a source of valuable nutrients, which are beneficial for long-term plant growth and productivity [47]. BS strain displayed higher root weight and reasonable root height. This indicates the accumulation of root biomass in BS-treated plants. When soil is incorporated with PGPR, it improves the growth parameters of plants with the excretions of several phytohormones in the rhizosphere, which has substantial endogenous control [48]. Previously, PGPR was helpful in the elongation of root cells by inducing indole acetic acid and stimulating the physiological responses of plants, which shows an increase in water and nutrient absorption [49, 50].

Soluble sugars are the essential substances that take part directly in metabolism by providing energy and building structure for plant cells. Our results showed a significant increase in the concentrations of soluble sugars in bacterized Sorghum plants (B26, BS, and BSER-treated plants) compared to controls (C− and C+) under saline (75 mM) and nonsaline conditions (0 mM). The increase in soluble sugar concentrations is strictly involved in abiotic stress tolerance. These sugars behave like osmoprotectants and signaling molecules in plants [51]. Under salt stress, the *Bacillus thuringiensis* (PM25), was previously reported to elevate maize plants' soluble sugars and protein content levels [52, 53]. Further, microbes-stimulated soluble sugars not only allow plants to withstand oxidative and osmotic stressors but also take part in improving plant growth and productivity [53]. Proline is an important amino acid playing a crucial role as an osmoprotectant in salinity stress tolerance. However, our results showed higher proline contents in control plants (C−) compared to other treatments. Further, biogas digestate-treated controls (C+) showed lesser proline contents in Sorghum plants. It is ascribed to an increase in the salinity stress conditions in the control plant compared to the other treatments. Increased proline content in salt stress conditions could maintain plants' sodium and K ratio [54].

Results of Secondary metabolite revealed that BS with BR and salinity showed the highest amount of phenols and flavonoid concentration in leaves of sorghum. Phenolic contents in plants are generally considered a crucial class of biochemicals responsible for plant stress resistance. Various researchers have reported elevated levels of phenolic contents in increased salinity levels in tissues of several plants [55]. Recently, Punia et al. [56] revealed that phenolic compounds in Sorghum displayed higher antioxidant activity in grains. Phenolic compounds are essential in inactivating lipid free radicals and preventing hydroperoxide decomposition into free radicals. Many types of PGPR including *Bacillus* species previously stimulated the production of phenolic compounds and the activities of enzymes linked to polyphenol synthesis including phenylalanine ammonia-lyase (PAL) and polyphenol oxidase (PPO) [14]. Our results showed that BS strain stimulated the production of polyphenol synthesis in Sorghum. That increases salinity tolerance by reducing oxidative damage in this specific situation; hence, plants showed higher growth than other treatments. Similarly, flavonoid contents in Sorghum also increase in bacterized plants, especially BS and B26. Gago et al. [57] revealed that flavonoids and phenolic content enhanced in halo-tolerant PGPR-treated plants result in the inactivation of reactive oxygen species (ROS) and lessen oxidative stress. An increase in the flavonoids in the leaves of plants alleviates the resistance against cellular damage against various stresses. The salt tolerance mechanism in cereals is closely related to producing flavonoids and other compounds that decrease oxidative damage [58]. Antioxidant enzymes provide stress tolerance via essential metabolic activity. Results indicating increased CAT activity in plants showed higher stress conditions. BS-treated plants exhibited lower CAT activities. Similar results were observed in the case of APX and SOD activities. PGPR-mediated decline of CAT activity has recently been reported in the Maize [59]. BR application demonstrated a significant decline in SOD, APX, and CAT activities, which can be correlated with improved plant growth and reduced salt stress in these treatments. Increased CAT and APX activities in stressed plants indicate higher production of free radicals in plant leaf tissues. However, GPX activities have increased in plants treated with BS and BR. This trend is opposite to the activities we observed in CAT, APX, and SOD. Figure 5 summarizes the trends of the parameters studied, indicating that growth parameters showed contrasting results to the antioxidant enzymes activities. We observed that the application of BR significantly improved the plant growth parameters when applied with bacteria, however, the enzymatic activities declined in the same treatments. Proline and phenolic contents showed a similar trend different from carbohydrates when BR were applied under saline conditions.

## 5. Conclusion

The combination of BR and *Bacillus* species was successfully processed to combat salinity stress, protecting plants from severe plant growth and yield losses. Plants treated with BR and BS strain demonstrate better plant growth than other *Bacillus* strains (B26 and BSER). Apart from growth, soluble sugars and proline were increased in sorghum plants treated with 75 mM salinity. Total phenolic compounds and flavonoids were higher when plants were treated with BS strain and BR. BR, along with BS strain, attained a friendly and sustainable way by reducing the use of chemical fertilizers to improve plant growth in salinity stress.

## Data Availability

Data will be available on request from the authors.
